# Combined endoscopic-percutaneous treatment of upper gastrointestinal enterocutaneous fistula using vacuum therapy and resorbable plug insertion (Vac-Plug)

**DOI:** 10.1038/s41598-022-15732-3

**Published:** 2022-07-18

**Authors:** Marcus Kantowski, Karl Karstens, Pasquale Scognamiglio, Nathaniel Melling, Matthias Reeh, Jakob Izbicki, Thomas Rösch, Michael Tachezy

**Affiliations:** 1 Department of Interdisciplinary Endoscopy, University Medical Center Hamburg Eppendorf, Hamburg, Germany; 2 Department of General, Visceral and Thoracic Surgery, University Medical Center Hamburg Eppendorf, Martini Str. 52, 20246 Hamburg, Germany; 3 Elisabethinum Medical Care Center, Hamburg, Germany

**Keywords:** Medical research, Outcomes research, Gastrointestinal cancer, Intestinal diseases, Oesophageal diseases, Stomach diseases, Ulcers

## Abstract

After gastrointestinal resections, leakages can occur, persist despite conventional therapy and result in enterocutaneous fistulae. We developed a combination method using flexible endoscopic techniques to seal the enteric orifice with an absorbable plug in addition to a percutaneously and fistuloscopically guided open-pore film drainage (Vac-Plug method). We retrospectively searched our endoscopy database to identify patients treated with the outlined technique. The clinical and pathological data were assessed, the method analyzed and characterized and the technical and clinical success determined. We identified 14 patients that were treated with the Vac-Plug method (4 females, 10 males with a mean age of 56 years, range 50–74). The patients were treated over a time period of 23 days (range 4–119) in between one to thirteen interventions (mean n = 5). One patient had to be excluded due to short follow-up after successful closure. Seventy-seven percent (10/13) were successfully treated with a median follow-up of 453 days (range 35–1246) thereafter. No treatment related complications occurred during the therapy. The data of the analysis showed that the Vac-Plug therapy is safe and successful in a relevant proportion of the patients. It is easy to learn and to apply and is well tolerated. In our opinion, it is a promising addition to the armamentarium of interventional methods of these difficult to treat patients. Of course, its usefulness must be further validated in larger prospective studies.

## Introduction

Leakages of gastrointestinal (GI) anastomoses and resection lines can result in acute and chronic entero-cutaneous fistula, leading to recurrent septic complications and possibly inadequate enteral nutrition. The optimal therapy of these fistulas is under debate, surgical solutions are often avoided due to the reduced general condition of the patients and the feared physical trauma of an early surgical re-intervention. Therefore, several conservative and especially interventional—endoscopic- approaches were developed during the last few years^1,2^. These methods include endoscopic vacuum therapy, stenting and closing procedures such as clipping, suturing, fibrin glue application, and absorbable plug insertion with variable success rates of up to 95%^2–4^.

In most parts of the world, the implantation of self-expandable metal stents (SEMS) is the gold standard in the therapy of esophageal and gastric leakages, but especially in older leaks (> 48 h old) a recurrent or permanent cleaning and drainage of the abscess cavity is required. This was addressed by Weidenhagen and colleagues, who established in 2003 the successful use of the endoscopic negative pressure therapy for treatment of rectal anastomotic leakages and later by Loske and colleagues in 2010 who had shown its benefit for upper GI leakages^5,6^. Recently, the method has been further developed, introducing open-pore film drainages, that can be placed intra- and extraluminally, even in cases of duodenal leakages^7,8^. Moreover, a placement through pre-existing drainage tracts was recently described in a pull-through method^9,10^. The advantage of the OPF lies in easy handling and good drainage capacity of GI fluids including biliary, duodenal, and gastric secretions^8^. A similar and more cost-effective method was recently described by de Moura and colleagues^11^. However, negative pressure might lead to discomfort, which is aggravated by limited oral intake (liquids only), and trans-esophageal or transnasal suction tubes. We developed a more convenient method avoiding the nasal tube by using the fistula or drainage tract as an access to the leakage site. Using flexible fistuloscopy, negative pressure therapy is applied transcutaneously with the recently described open-pore film drainage^8^. The second step consists of closure of the orifice with an absorbable and water-proof plug. The idea of endoscopic or fistuloscopic plug insertion in fistulas and leakages has already been described by Pross and colleagues in 2000 using a cylindric Vicryl plug combined with fibrin glue and later a Vicryl mesh plug^12,13^. In 2008 Toussaint and colleagues implanted porcine small intestine plugs, designed for the treatment of anal fistulas, in five patients with entero-cutaneous fistula after bariatric surgery for the first time using a rendezvous-method. Later, the same method was described in a larger series of upper GI fistulas by Filgate and colleagues (n = 14)^14,15^. The authors described success rates of two out of five (40%) and 13 out of 14 (93%), respectively.

By combining transcutaneous suction and plugging of the orifice (Vac-plug method) we expect the following benefits: negative pressure therapy should ideally hold the plug in the correct position while actively evacuating wound liquids and enteral fluids in case of incomplete closure. In addition, reduction of fistula size over time will facilitate a better incorporation of the plug in the surrounding tissue. Of note, patients can start an oral diet and do not need a per-nasal suction tube, which impairs quality of life. We present our results of a series of 14 patients treated with this method.

## Materials and methods

### Patients and clinical data

A retrospective search was performed of our endoscopic database 2017–2020, using the search terms ‘Plug’ AND ‘Vacuum therapy’. Cases were selected with the following criteria:

1. confirmed upper—GI anastomotic or staple line leakage with a direct or drainage tube based entero-athmospheric or -cutaneous fistula,
2. patients were pre-treated with surgery (including vacuum therapy) and/or established endoscopic methods such as endoscopic vacuum therapy.

The study was approved by the Medical Ethical Committee Hamburg, Germany (approval number: PV3548). Informed consent was obtained from all patients before study inclusion. This retrospective study was performed in accordance with the ethical standards laid down in the 1964 Declaration of Helsinki and its later amendments and the local legal regulations (HmbKHG). All relevant clinical data regarding patients and endoscopic examinations were obtained from a combination of in- and out-patient clinical and endoscopic record reviews, and communication with patients and their attending physicians. The data obtained included healing of fistula, septic complications, and therapy-related complications.

### Technique of the Vac-Plug method

**Figure 1:**
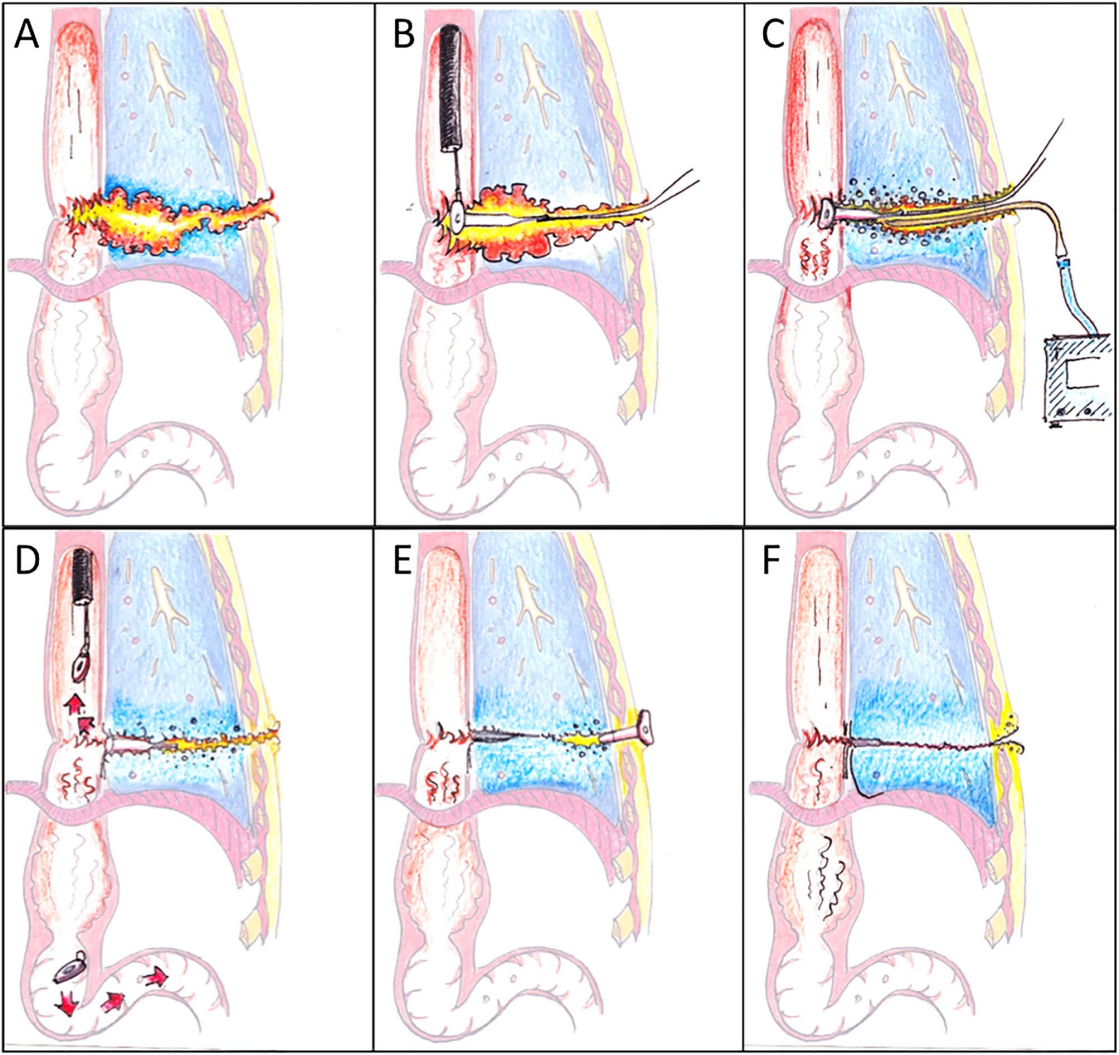
(**A**) Anastomotic leak with a percutaneous fistula. (**B**) Endoscopic plug implantation into the fistula orifice. (**C**) Percutaneous and fistuloscopically placed under pressure therapy with open-pore film drainage. (**D**) Separation of the plug plate and continuation of the vacuum therapy until closure of the orifice. (**E**) Avoidance of premature skin closure by implantation of a shortened und inverted PEG tube. (**F**) Removal of the PEG and final state.

The principle of our method consists of a peroral endoscopic plugging of the remaining orifice of the leak with an absorbable and ingrowing plug and percutaneous treatment of the remaining fistula and abscess cavity with endoscopic open-pore film drainage therapy (Fig. 1).

#### Plug preparation

**Figure 2:**
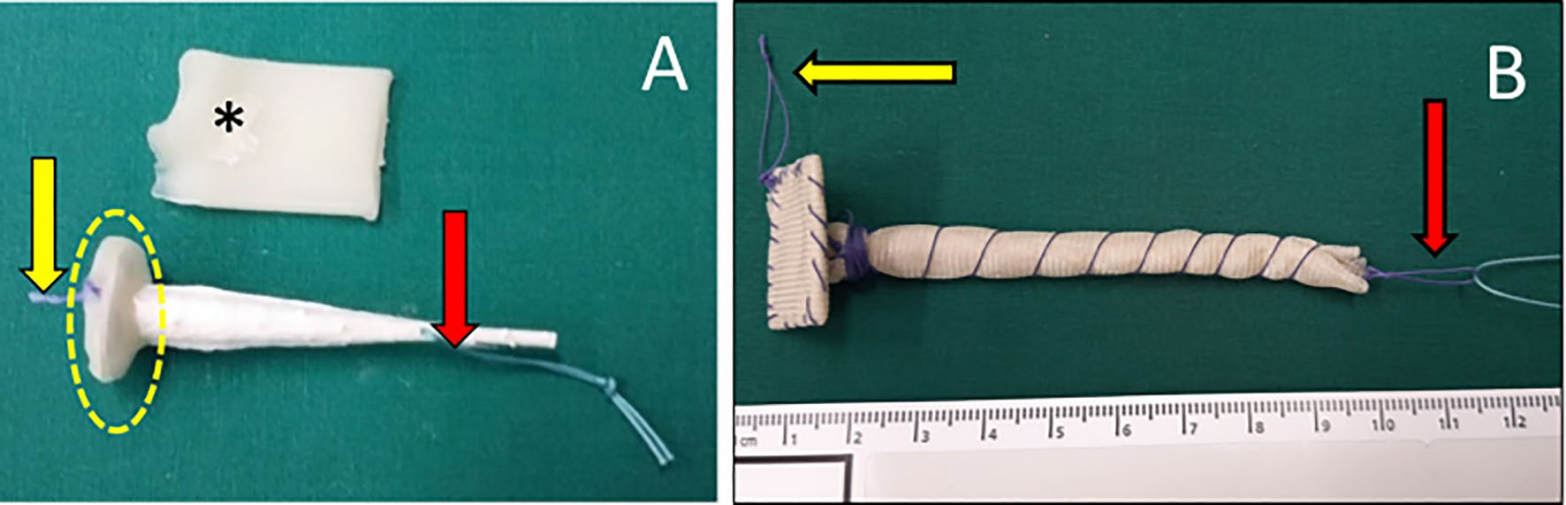
Two different types of plugs. (**A**) A modified Biodesign Fistula Plug (Cook), (**B**) self-made plug with waterproof cap and resorbable cone by Vicryl mesh. The base plate is sealed with bone wax (*). For endoscopic implantation, a strap with an absorbable suture is sewed inside the waterproof basis plate (yellow arrow) that can be grabbed by the endoscopic forceps and one strap at the tip of the plug (red arrow).

The plugs applied were either Biodesign Fistula Plugs (COOK Medical, 52,499 Baesweiler Germany) with an average degradation time of 14 days (Fig. 2A) or a self-made plug based on a Vicryl mesh (Polyglactin 910) copolymer of lactide (a cyclic diester of lactic acid) and glycolide (a cyclic diester of glycolic acid, Ethicon, 22,851 Norderstedt, Germany) with a slower degradation of approximately 90 days (Fig. 2B). The mesh was cut in two parts and a base plate and a cone were fashioned and sewed with absorbable sutures (Vicryl 3,0 metrics, Ethicon, 22,851 Norderstedt, Germany).

The plugs were then further modified before implantation: the plug was covered with a thin layer of Bonewax (Ethicon, 22,851 Norderstedt, Germany), which is a mixture of vaseline and beeswax (Fig. 2A, yellow dotted line). This creates a waterproof layer on the intra-luminal base-plate of the plug and prolongs the time of absorption and destruction by enteric fluids. Without this procedure the porcine mucosa plug would be digested and destabilized in the upper GI tract within 2–3 days. For endoscopic implantation, a strap with an absorbable suture was sewn onto the inside of the waterproof base, so it could be grabbed by the endoscopic forceps. Furthermore, one strap was fixed at the tip of the plug (Vicryl 3,0 metrics, Ethicon, 22,851 Norderstedt, Germany, Fig. 2A, yellow and red arrow). A second Vicryl loop (about 75 cm long) was knotted to the strap at the tip for the pull through maneuver and later served as an external fixation to the skin so as to avoid plug dislocation on the intestinal side.

### Plug insertion

First a suture is pulled through the fistula into the enteric lumen and then externalized through the mouth: Fistuloscopy is performed with a small lumen endoscope (such as GIF-XP190N, Olympus Corp., Tokyo, Japan) and a guide wire (Jagwire; Boston Scientific, Natick, MA) is placed through the leakage or through a drain, if still in place. Via peroral endoscopy, the end of the guide wire is grasped with a snare or forceps and externalized. Over the guide wire, an endoscopic retrograde cholangiopancreatography catheter is introduced and the guide wire replaced. A thick monofilament suture (e.g. PDS 1,0 metrics, Ethicon, 22,851 Norderstedt, Germany) is pushed through the whole length of this catheter. The catheter is then removed and the suture is provisionally fixed to the skin and outside the mouth. At this time drainages can be removed. Endoscopic flushing and debridement of the fistula is performed. In some cases, an endoscopic fistula brush might help in the process (Fistula Brush PR, OVESCO, Tuebingen, Germany).

**Figure 3:**
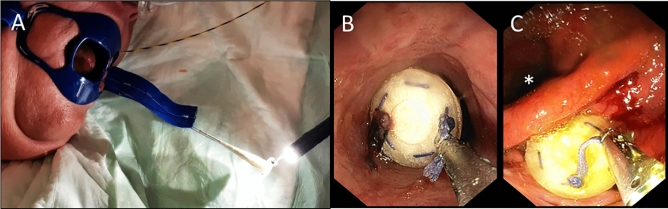
(**A**) The loop at the base plate of the plug is grasped. (**B**) By careful pulling of the suture that is fixated at the tip of the plug and is externalized through the fistula to the skin, (**C**) the plug is placed at the enteric fistula orifice under endoscopic vison.

If the fine caliber endoscope cannot be pushed through the orifice, a dilation with a balloon catheter is performed. Another option is to omit the fistuloscopic exploration and to introduce of a thick monofilament suture (e.g. PDS 1,0 metrics, Ethicon, 22,851 Norderstedt, Germany) via the drainage to pull it with an endoscopic forceps through the orifice. The second strap on the tip is connected with the transoral suture and the plug is now endoscopically pulled through to the intestinal orifice (leakage) (Fig. 3A,B) with a forceps (Fig. 3C). After exact positioning of the plug, the long strap knotted to the suture at the tip of the plug is fixed to the skin to prevent intraluminal dislocation of the plug. The external loop can be removed 10 days after plug implantation with only little risk of dislocation.

#### Percutaneous suction therapy

Depending on the size and the depth of the fistula, an open pore film drainage is used to stimulate the ingrowth of the plug by suction and evacuation of secretions and debris. Also, it reduces the size of the defect by formation of granulation tissue.

**Figure 4:**
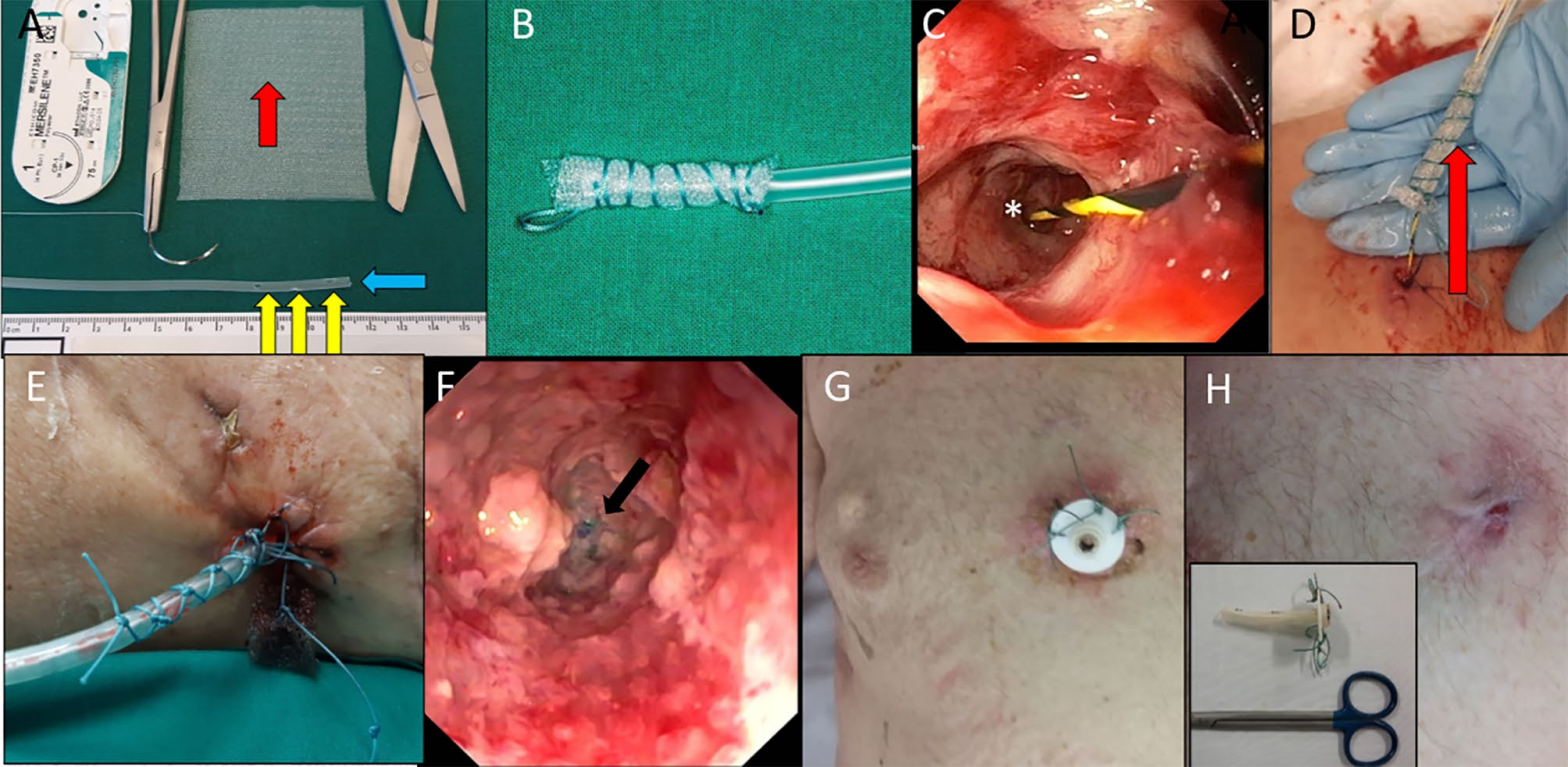
(**A**) A small diameter catheter prepared with several side- (yellow arrows) and end whole (blue arrow) is covered with a segment of open-pore film (red arrow, Suprasorb-CNP, Lohmann & Rauscher, 56,579 Rengsdorf, Germany). (**B**) Open-pore film drainages in (12 Charrière, 4 cm length). (**C**) The tip (*) of a guide wire is placed on the ground of the fistula tract. (**D**) After fistuloscopic measurement of the length with a small caliber endoscope, the open-pore film drainage is placed inside and externally fixed with sutures (**E**). Fistuloscopic image after plugging and 10 days of open-pore film therapy. The tip of the plug (*) is still visible but seems to be ingrowth. (**F**) In case of a long fistula tract, a PEG is inserted in the fistula/abscess for daily rinsing (**G**). After fistuloscopic control, the PEG can be further shortened or removed (**H**).

A small diameter catheter (Fig. 4A, yellow arrows) covered with an open pore film (Suprasorb-CNP, Lohmann & Rauscher, 56,579 Rengsdorf, Germany, Fig. 4A) is inserted into the distal fistula tract between the tip of the plug and the skin, and continuous suction is applied using an electric pump (ActiV.A.C, setting: – 125 mmHg, continuous negative pressure, high intensity; KCI, San Antonio, Texas, USA—Fig. 4B). This procedure too, is performed using a guide wire, with the tip placed close to the end of the plug (Fig. 4C,D). The distance between the skin and the plug should be measured with a small caliber flexible endoscope beforehand. Avoiding a dislocation, the tube is stitched on the skin (Fig. 4E).

#### Changing process

Exchange of the suction material can safely be performed every 3–7 days over a guide wire under fistuloscopic and endoscopic control with the small diameter endoscope (Fig. 4F). Compared to the sponge, the open-pore film drainage allows a longer duration of suction (up to seven days) since the ingrowth is reduced and the film is not as fragile as the sponge. Status of closure, granulation and contamination should be evaluated during endoscopy. Also, the degradation process of the plug should be monitored.

The fistula tract is rinsed, remaining necrosis, leftovers or foreign bodies are removed. The size and length of the newly placed open-pore film drainage should be adapted to the decreasing size of the fistula/abscess.

Percutaneous suction therapy should be stopped when the tract between the plug and the skin is short enough for spontaneous healing and closure of the intestinal orifice is reached. In cases of a long remaining fistula tract, we placed in some cases a shortened PEG tube with the gastral plate placed at the skin into the remaining tract for daily rinsing to avoid a to early closure of the skin with the risk of a recurrent abscess (Fig. 4G). After further wound healing, the PEG tube can be shortened or later omitted (Fig. 4H).

### Statistical analysis

Due to the exploratory nature of this pilot series, no comparative data were analyzed. All statistical analyses were carried out using IBM SPSS Statistics for Mac (Version 20, IBM Corporation, Armonk, New York, USA). The median and range were used to describe the results.

## Results

Between April 2017 and December 2020, 14 patients with postoperative upper GI fistulas were treated with endoscopic plug insertion and percutaneous suction therapy at the University Hospital Hamburg-Eppendorf. Patient characteristics regarding age, sex, underlying disease, and initial surgery performed are listed in Table 1.

**Table 1:** Patients characteristics.

Pat number	Sex	Age	Disease	Surgical procedure	Fistula	Anastomosis	Abdomen apertum	Size of the leakage (cm)	Fistula length (cm)	Primary leakage therapy	Previous endoscopic therapy	Time between initial Surgery and Leakage diagnosis (days)
1	m	63	Esophageal Cancer (Adeno)	Minimally invasive Ivor Lewis Esophagectomy	Esophago-pleural-cutaneous	Esophago-gastrostomy	No	0.5	10.0	Endoscopic	EVT, Fibrin glue	2
2	m	56	Esophageal Cancer (Adeno)	Minimally invasive Ivor Lewis Esophagectomy	Esophago-pleural-cutaneous	Esophago-gastrostomy	No	0.5	22.0	Endoscopic	EVT, Stenting	5
3	m	60	Esophageal Cancer (Adeno)	Minimally invasive Ivor Lewis Esophagectomy	Esophago-pleural-cutaneous	Esophago-gastrostomy	No	0.5	14.0	Endoscopic	EVT	15
4	m	51	Esophageal GIST	Ivor Lewis Esophagectomy, Colon conduit	Esophago-pleural-cutaneous	Esophago-gastrostomy	No	0.8	12.0	Endoscopic	EVT	10
5	m	55	Esophageal Cancer (Adeno)	Ivor Lewis Esophagectomy	Esophago-pleural-cutaneous	Esophago-gastrostomy	No	1.0	13.0	Endoscopic	EVT, Stenting	12
6	w	49	Esophageal Cancer (Adeno)	Robotic Ivor Lewis Esophagectomy	Tracheo-esophago-pleural-cutaneous	Esophago-gastrostomy	No	0.3	22.0	Surgery	EVT	12
7	m	67	Achalasia/ Esophago-tracheal fistula	Esophago-bronchial Fistula after Ivor Lewis Esophagectomy, Colon conduit	Gastro-pleural-cutaneous	Esophago-colostomy	No	0.3	23.0	Surgery	EVT	1157
8	m	74	Chronic pancreatiis	Pancreatectomy, total Gastrectomy	Esophago-abdominal-cutaneous	Esophago-jejunostomy	No	1.5	20.0	Endoscopic	EVT	18
9	w	50	Morbid Obesity	Sleeve gastrectomy	Gastro-cutaneous	–	No	0.5	35.0	Surgery	EVT	10
10	m	52	Morbid Obesity	Sleeve gastrectomy	Gastro-cutaneous	–	No	0.5	25.0	Surgery	EVT, Clipping	7
11	w	48	Morbid Obesity	Sleeve gastrectomy	Gastro-peritoneal	–	Yes	1.0	3.0	Endoscopic	EVT	5
12	m	66	Colorectal mesenterial metastases	Liver resection, tangential duodenal resection	Duodeno-peritoneal	Duodeno-jejunostomy	Yes	3.0	10.0	Surgery	EVT	41
13	w	69	Colorectal mesenterial and liver metastases	Liver resection, pars IV duodenal resection	Gastro-abdominal-cutaneous	Gastro-enterostomy	No	0.5	20.0	Surgery	EVT	21
14	m	53	Colorectal mesenterial and liver metastases	Liver resection, pars IV duodenal resection	Duodeno-peritoneal	Duodeno-jejunostomy	No	0.5	12.0	Surgery	EVT	21

### Treatment details and duration

The time required for the endoscopic evaluation as well as application of plug and suction varied significantly depending on the location and the size of defect. The first procedure with plug and open-pore film drainage placement took a median of 47 min (range 32–118 min). However, the following endoscopic explorations for reevaluation and exchange of the suction material took significantly less time (usually 12–48 min).

In median, combined plug and vacuum therapy started 72 days after initial surgery (range 12–1282 days) and lasted for a median of 23 days (range 4–119 days) with a median of five endoscopic interventions (range 1–13). Length of therapy was dependent on size and complexity (branches and cavities) of the fistula. For example, one patient (No 9) presented with a branched fistula which remained undetected until development of a further abscess, which was opened and treated with a second open-pore film catheter prolonging the therapy for another 12 days. Twelve fistula plugs and two Vicryl plugs were used (Table 2).

**Table 2:** Results and comparison of the treatment success (One patient was excluded due to short follow-up).

		Leakage closure	
		Yes (n = 10 pat., 77%)	No (n = 3 pat., 23%)
Median time between surgery and start of the Plug-Vac therapy (days)	Median (range)	60 (12–385)	97 (30–1282)
Size of the fistula orifice (cm)	Median (range)	1.0 (0.5–1.5)	1.0 (0.3–3.9)
Lenght of the fistula (cm)	Median (range)	20 (10–35)	10 (3–10)
Duration of the Vac-Plug therapy (days)	Median (range)	23 (15–119)	68 (4–75)
Vicryl™ Plug (n)	Number	2/10 (20%)	0/3 (0%)
Number of Vac-plug interventions (n)	Median (range)	5 (1–13)	9 (1–12)
Abdomen apertum (n)	Yes	0 (0%)	2 (67%)
Primary transmural defect therapy	Endoscopy	5	1
	Surgery	5	2
Lenght of hospitalization (days)	Median (range)	84 (4–160)	141 (90–338)
Mortality	Yes	0 (0%)	3 (100%)

No therapy-related complications were seen during the Vac-Plug therapy. Patients were hospitalized for a median of 92 days (range 4–338 days). Of note, three patients were treated with the transcutaneous placed open pore-film drainage and electronic vacuum device on an outpatient basis. Patient acceptance of the method was good since trans-nasal suction tubes could be omitted due to the percutaneous suction.

### Closure rates and complications

Nine patients were treated successfully and the fistulas closed without any further clinically relevant findings and we had a sufficient follow-up for a healing process (453 days (range 35–1246)). In one patient (No 6), the fistula was technically successful treated, but due to a long-lasting esophageal fistula and systemic tumor recurrence, she died of pneumonia 10 days after the end of the therapy; therefore, the therapeutic success could not be fully evaluated. One patient (No 7) did not show any significant healing progress. The infection was maintained by an intra-thoracic non resorbable mesh and a modified PEG was inserted to ensure proper drainage. The patient died nine days later following acute septic portal vein thrombosis. In two patients (No. 11 and 12) the fistula persisted resulting in recurrent septic complications (open abdomen and huge leakage of 3 cm in diameter) so that surgical revision was performed. However, in both cases another leakage re-occurred and the patients died due to septic complications. Of note, one clinically relevant esophageal stenosis and a duodenal stenosis were detected in the late course requiring surgical and endoscopic intervention. Thus, success rate with adequate follow-up was 10/13 cases (77%).

## Discussion

In this study, we present our experience of 14 patients treated with an innovative method of fistuloscopically applied negative pressure therapy combined with an endoscopic plugging of the intestinal orifice with an absorbable fistula plug. As already described, different methods of plugging were presented during the last years, but the combination with the negative pressure therapy might substantially increase the success rate. As a result of the negative pressure therapy, the formation of granulation tissue inside the fistula starts, the tissue collapses, the plug remains in place and a sealing with the plug can be achieved. Another innovative aspect is the percutaneous application to omit a trans-nasal suction catheter, that is described as very disturbing by the majority of patients.

The technique was applied as a second- or third-line therapy in endoscopically pre-treated upper GI enterocutaneous fistula of various origins and underlying diseases, predominantly after esophageal and gastric resections (Table 1). The endoscopic access to the leakage via the fistula’s cutaneous orifice was often straight and short allowing a much easier way to access the region of interest than the usual endoscopic approach. In contrast to the vacuum sponge therapy, we experienced far less ingrowth into the surrounding tissue with open-pore film drainage thus allowing for much less atraumatic replacement of the suction material. Of note, we did not observe any severe complications associated to the treatment applied, such as bleeding or septic complications caused by a dysfunctional system or a delayed replacement. This is in accordance with the observations made by Loske et al.^8^. Hence, the method appears to be safe and easy to learn.

In 79% or eleven of the 14 enrolled patients the leakage was technically sealed. The three (21%) remaining patients were treated unsuccessfully. In one patient (number 7) the fistula was very long (23 cm) and showed hardly any ingrowth of the tissue surrounding the plug. Hence, healing was not observed. The other two cases posed technical problems due to large (1 cm and 3 cm, respectively) intestinal orifices and open abdomen with a rather short fistula tract (3 cm and 10 cm, respectively).

The results of our series demonstrate, that transcutaneous/ fistuloscopic endoscopic vacuum therapy can be used as an alternative option to transoral endoscopic vacuum therapy. However, the leakage site can only be reached by a preexisting transcutaneous drainage or an established fistula tract so as to allow sufficient endoscopic visualization. Application of a plug into the intestinal orifice can sufficiently occlude the leakage. In the presented technique, the closure of the leakage is supported by the continuous negative-pressure therapy, which induces a permanent sealing of the leak by stimulation of tissue growth. We were also able to improve the already described plugging methods in our technique by sealing of the endoluminal base-plate with bone-wax to reduce contact to the enteric fluids and subsequent degradation of the plug^12–15^. In our experience, without this cover the porcine mucosal plug would be destabilized and partially resorbed within two or three days. In addition to the commercially available plugs, we developed a self-manufactured vicryl net plug, which revealed improved stability and was variable in length and size. These individually customized plugs are useful in patients with larger defects and longer fistulas, in which anal fistula plugs are too small to close the orifice. Moreover, the surface of the vicryl plug is rougher and more fluid-permeable for a faster tissue ingrowth. More patients need to be treated with the vicryl plug to make some solid conclusions if its superior to the porcine mucosal plug.

Another alternative method could be a combination of endoscopic implantation of self-expanding metal stents (SEMS) with percutaneous suction therapy. This allows intraluminal closure of the leak and early peroral nutrition. However, the well-known drawbacks of stenting like the risk of stent dislocation, bleeding, perforation and ingrowth of the non-coated part as well as a relatively high costs remain^3^. Moreover, a sufficient sealing of the leakage is not always possible due to divergence of the lumen, while plug application can be performed even in intricated situations and with incongruent enteric lumen^16^. A method that combines the sealing of a stent and the benefits of the negative pressure therapy is VAC-Stent or Stent-over-Sponge (SOS) method^17,18^. The patients can start the per-oral nutrition early, but a nasal suction tube still remains necessary.

Another group described polyglycolic acid sheets combined with fibrin glue for esophageal fistulas following esophagectomies in a series of five patients with a reported closure rate of 40%^19^. Further closure methods combine anal fistula plug insertion and mucosal covering with endoscopic sutures using the Apollo overstitch system, as just recently described in a video case report^20^. High costs and difficult maneuvering of the suture device in a narrow and fixed defect might be a limiting factor in the usage of this procedure. Difficult anatomy, funnel-shaped opening of the leakages or differences of the diameter between the intestinal organs often cause problems of occlusion by GI stenting, OTSC clipping or endoscopic suture. In these cases, the Vac-plug method might be a useful alternative. Another method to close the orifice of chronic fistula is the off-label insertion of cardiac septal occluders, that showed a good clinical effect in a series of post-bariatric surgery fistulas, but the relatively high price of the device must be considered^21,22^.

Based on our experience, the best time to apply our method is just after diagnosis of the fistula. In the early phase of the leakage, a short endoscopic pre-treatment with conventional transluminal negative-pressure therapy (sponge or open-pore film) might be useful to clean and stabilize the tissue, initiate granulation and await the final extent of the intestinal orifice. Notably, a vacuum therapy is indicated only if a negative pressure can be achieved. In our opinion, in stable patients a treatment attempt is justified even for older leakages since the complication rate is rather low. If the vac-plug therapy fails, other, more invasive, treatments, such as surgical revision, can still be performed.

Flexible endoscopic transcutaneous debridement, rinsing and usage of sponge or open-pore film drainage therapies, as described, could be a promising option in septic diseases and postoperative complications when no contact to the enteric lumen exists. Such abscesses or even pleural empyema, that are usually drained surgically could be ideal cases for this technique^23^.

The general drawbacks of combining intraluminal plug and percutaneous suction therapy are similar to those of transluminal sponge or open-pore film therapy: Costs are fairly high due to the expensive materials used, the need for a small caliber endoscope, and repeated endoscopic interventions with the need of sedation. These sedations might also have an impact on the patients’ quality of life including a general procedure and sedation related risk^24^. On the other hand, the method can be used on an outpatient basis and the patients can start per-oral nutrition. This might lead to an earlier anabolic nutritional status, which again facilitates wound healing.

This study has several limitations due to the small number of patients including and the lack of a comparison group. It is a prospective observational study to prove the feasibility and safety of the concept and further studies must show its broader clinical applicability and potential superiority over other methods.

In summary, the combination of intraluminal plug therapy and percutaneous negative-pressure therapy might be a promising additional tool in the therapy of upper GI leakages. It can be used in both early and persisting fistulas and might be an option even in refractory cases since the peri-interventional risk is low.

The data of the manuscript were presented at the annual interdisciplinary meeting ‘Visceral Medicine’ in 09/2021 and online published as an abstract in the journal ‘Zeitschrift für Gastroenterologie’ (Z Gastroenterol 2021; 59(08): e291-e292).

## Data Availability

The datasets generated during and/or analyzed during the current study are available from the corresponding author on reasonable request.
